# Heparin Binding Proteins as Therapeutic Target: An Historical Account and Current Trends

**DOI:** 10.3390/medicines6030080

**Published:** 2019-07-29

**Authors:** Giancarlo Ghiselli

**Affiliations:** Independent Researcher, 1326 Spruce Street Suite 706, Philadephia, PA 19107, USA; gghiselli@meetsci.com

**Keywords:** sulfated glycosaminoglycans, heparin-binding proteins, fibroblast growth factor, chemokines, xylosides, sulfotransferase inhibitors, heparanase, small molecule drugs

## Abstract

The polyanionic nature and the ability to interact with proteins with different affinities are properties of sulfated glycosaminoglycans (GAGs) that determine their biological function. In designing drugs affecting the interaction of proteins with GAGs the challenge has been to generate agents with high binding specificity. The example to emulated has been a heparin-derived pentasaccharide that binds to antithrombin-III with high affinity. However, the portability of this model to other biological situations is questioned on several accounts. Because of their structural flexibility, oligosaccharides with different sulfation and uronic acid conformation can display the same binding proficiency to different proteins and produce comparable biological effects. This circumstance represents a formidable obstacle to the design of drugs based on the heparin scaffold. The conceptual framework discussed in this article is that through a direct intervention on the heparin-binding functionality of proteins is possible to achieve a high degree of action specificity. This objective is currently pursued through two strategies. The first makes use of small molecules for which in the text we provide examples from past and present literature concerning angiogenic factors and enzymes. The second approach entails the mutagenesis of the GAG-binding site of proteins as a means to generate a new class of biologics of therapeutic interest.

## 1. Introduction

Sulfated glucosaminoglycans and galactosaminoglycans (GAGs) gain biological relevance by binding and modulating the function of proteins [1,2,3,4]. Their polyanionic nature is however limiting the interaction specificity, and GAGs may bind indiscriminately to different proteins supporting a vast range of biological events [5,6,7]. The presence of GAG-neutralizing proteins restricts the range and regulates these events [8]. Phylogenetically, the advent of GAGs anticipates that of proteins and of biological circuitries that in mammals are strictly dependent on GAGs for their function. For example, heparin extracted from mollusks activates anti-thrombin III (AT-III) and has anticoagulant activity comparable to that of mammalian heparin [9] in spite the fact that these animals lack a functional coagulation system. Proteins may have acquired the ability to interact with GAG in order to gain specialized functions in circumstances in which the physicochemical properties and the signature structure of GAGs have remained virtually unchanged [10]. 

From a therapeutic point of view, heparin has been the most investigated GAG. Its binding avidity to proteins (referred to as heparin-binding proteins) represents the benchmark against which the activity of other GAGs is compared [11]. Parenteral heparin has huge medical importance as anticoagulant and anti-thrombotic agent and together with its antidote, protamine sulfate, and fragmented low-molecular weight heparin (LMWH) is listed as essential medicines by the World Health Organization [12,13,14]. A fully synthetic pentasaccharide (Fondaparinux-Arixtra) corresponding to the AT-III interacting domain of heparin is available for clinical use [15,16]. In the vessels, heparan sulfate (HS), which is structurally related to heparin, performs the role as resident anti-thrombotic agents with the potential to activate AT-III and other factors of the coagulation cascade. Circulating proteins and those that are present at the cell surface interact with HS, and the encounters have important biological consequences that go beyond those linked to the coagulation and thrombosis systems [11,17,18,19]. The interaction between proteins with HS has been the subject of intense investigation during the past decades [20,21,22] but the attempts to identify structural determinants in HS that can support specific interactions with proteins have not matched the expectations set forth by the heparin/AT-III lock-and-key binding paradigm. 

In response to the lack of significant breakthroughs, new lines of research have been initiated that regard the heparin-binding proteins as a target for drug design in alternative to the interacting polysaccharides. The finding that blockade or modification of the heparin binding domain in proteins significantly affects their biological activity has contributed to setting this trend in motion. The neutralization of the heparin binding site in proteins by small molecules is emerging as an effective strategy to modulate the activity of angiogenic factors and enzymes. The inactivation or the amplification by mutation of the heparin binding site in chemokines and other proteins is a further recent development in the field that is being exploited to generate a new class of biological drugs. This review will focus on these promising new developments, will discuss them in terms of their potential for drug development and will further consider what new information on GAG biology has emerged from these studies.

## 2. Heparin Binding Growth Factors and Their Inhibitors 

**Figure 1:**
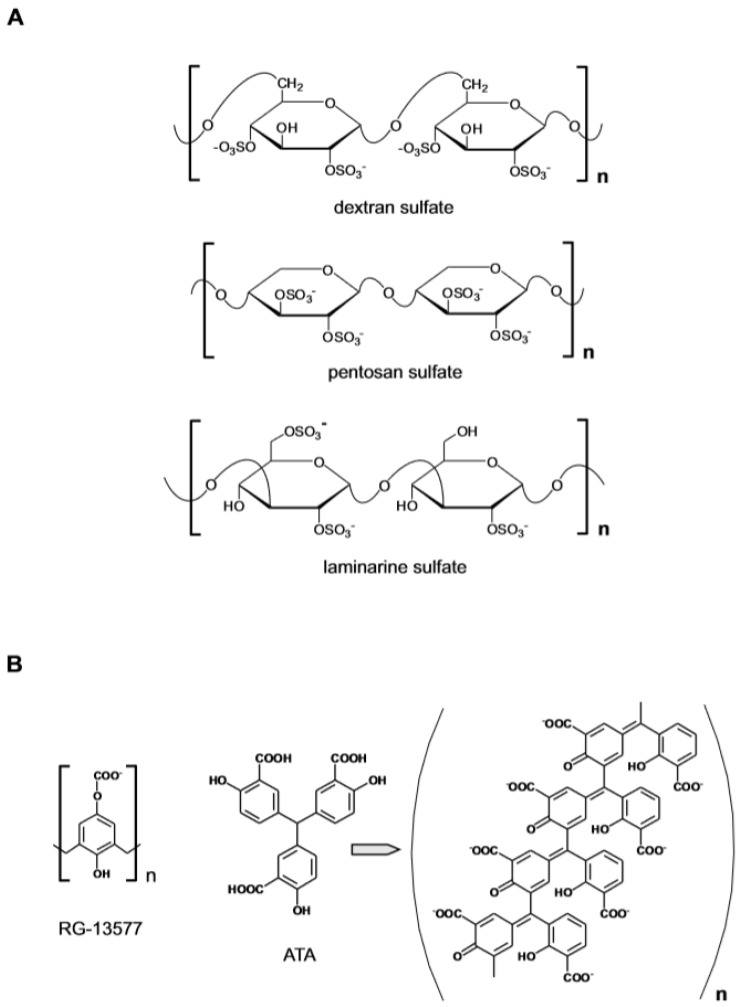
Anionic polymers as heparin-mimetic. (**A**) Polysulfated polymers based on a carbohydrate backbone. (**B**) Fully synthetic polycarboxylic polymers.

The growth factors FGF1, FGF2 and VEGF are important mediators of angiogenesis that rely on the interaction with cell surface HS to productively engage cell membrane receptors to initiate signal transduction [23,24]. Evidence of a functional interaction between FGF and heparin was presented in 1983 when it was reported that at low concentration heparin enhanced the effect of an endothelial cell growth factor (a crude preparation of FGF1), enabling the maintenance in culture of endothelial cells from adult human blood vessels [25]. The effect of heparin could be mimicked by the synthetic sulfated polysaccharide dextrane sulfate (Figure 1A), whereas other extractive GAGs such as chondroitin sulfate/dermatan sulfate (CS/DS), keratan sulfate and hyaluronic acid had no effect. The observation that heparin in addition to interacting with FGF also binds to a specific domain of the tyrosine kinase FGF receptor (FGFR) led to the definition of the molecular mechanism involving HS in angiogenesis [26]. It was thus established that the formation of the tripartite complex between FGF, FGFR and HS is required for the activation the angiogenic FGF-dependent signaling pathway. This conceptual framework has influenced subsequent attempts to identify specific modulators of the process and to develop anti-angiogenic agents. 

The model predicted that an anti-angiogenic effect could be achieved if heparin/HS or one of the surrogates was present at high concentration due to the ability of the sulfated polysaccharides to sequester the angiogenic factor by competing with HS at the cell surface. An example of this mode of action was provided by a study in which the administration of the synthetic polymer pentosan polysulfate (MW ~5.7 KDa) inhibited angiogenesis and the growth of subcutaneous human tumor xenografts in nude mice [27]. On the other hand, at low concentrations, the heparin mimetics could induce an angiogenic response by replacing the cell resident HS in the assembly of the FGF–FGFR signaling complex. Such was the case of the semi-synthetic branched poly 1,3 glucan-sulfate polysaccharide (laminarine) which could activate the FGF mitogenic pathway in GAG-deficient CHO cells and in HS-deficient lymphoid cells [28]. Among non-sulfated polyanionic polymers (Figure 1B), RG-13577 (a co-polymer of 4-hydroxyphenoxyacetic acid and formaldehyde ammonium salt, MW ~ 58 KDa) inhibited the mitogenic activity of FGF2 by directly abrogating the heparin-mediated dimerization of FGF2 and FGFR1 [29]. The polymeric aurin tricarboxylic acid (ATA) instead exerted anti-angiogenic activity by binding to FGF2 and by altering the polypeptide conformation [30]. Collectively the results demonstrated that the polyanionic nature of GAG had primary relevance for their biological activity and that different effects could be produced. No strict structure–function relationship however emerged that could inspire the rational design of specific agents. 

**Figure 2:**
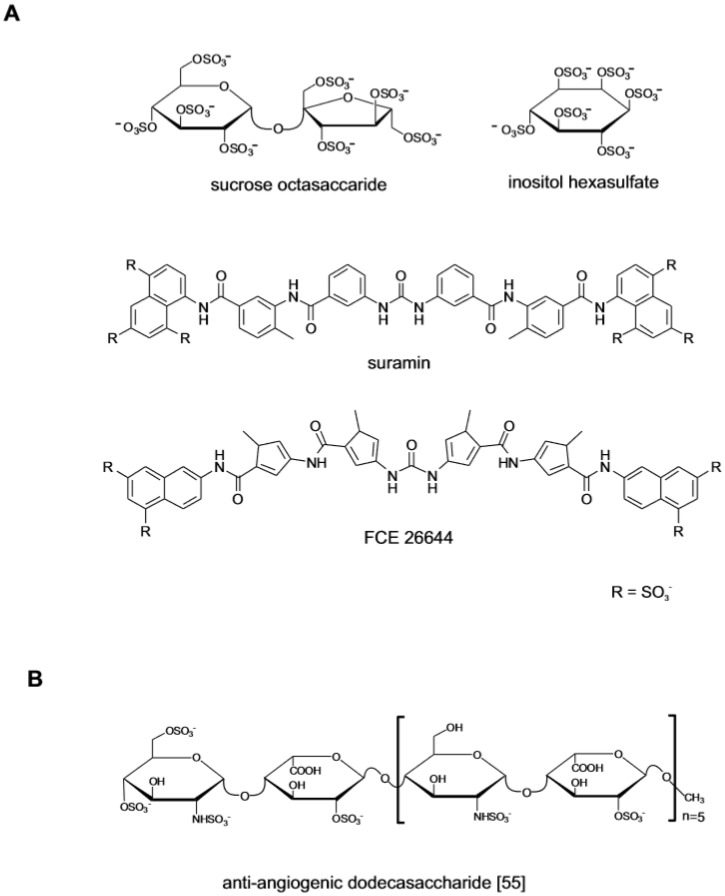
Small molecule FGF ligands and rationally designed oligosaccharides. (**A**) Early investigated polysulfated small molecule drugs with anti-angiogenic activity. (**B**) Structurally defined heparin-like dodecasaccharide generated through a completely synthetic process. Structure redrawn from ref. [55].

At the same time that the studies on polymeric compounds were completed, polysufated small molecules also attracted interest as FGF ligands (Figure 2A). The anti-ulcer drug sucrose octasulfate (SOS) was investigated because its structure, a disaccharide with eight sulfate residues, resembled the repeating disaccharide units of heparin [31]. The crystallographic analysis of the SOS in complex with FGF1 showed that the molecule binds to FGF1 and FGF2 at the same site as that of heparin protecting the growth factor from degradation in the upper gastric tract [32,33]. The monosaccharide inositol hexasulfate (MIHS) was also identified as a ligand of the heparin-binding site of FGF1. Binding to MIHS changes the conformation of the growth factor, enhancing its interaction with FGFR and promoting signaling [34]. The attempts to identify non-carbohydrate small molecule with anti-angiogenic activity centered on suramin, a highly sulfated small molecule [35]. While undergoing clinical trial in HIV patients, it was observed that although failing as anti-viral, suramin decreased the incidence of HIV-related lymphoma and Kaposi sarcoma in the treated patients [36]. This lead to its investigation as anti-tumor agent focusing on the interaction with heparin-binding growth factors [37,38]. The drug and the structurally related suradistas (sulfonic distamycin-A derivatives) such as compound FCE 26644, bind to FGF2 with high affinity. By inhibiting the interaction between FGFs and the cognate cell surface receptors, these compounds abrogate the mitogenic and the angiogenic response [39,40]. Due to off-target effects and a penalizing toxicological profile, suramin and its analogues could not be developed clinically [41].

These early studies defined the chemical space within which heparin-mimetics could be identified and offered clues on the pharmacological mechanisms that could be exploited to modulate angiogenesis. Since then new layers of complexity have been added to the heparin–FGF–FGFR interaction model [42,43,44,45,46]. The supporting medicinal chemistry has progressed along two lines. The first has focused on structurally defined heparin-like oligosaccharide based on the idea that by tailoring their sulfation pattern it is possible to specifically target different growth factors. This approach has received impulse by the development of procedures to generate oligosaccharides with defined structure. The second line of investigation has instead centered on a small non-carbohydrate molecules capable of interacting with the heparin-binding site of the angiogenic factors. The optimization of their activity has taken advantage of consolidated medicinal chemistry practices that are difficult to implement in heparin-like oligosaccharides.

### 2.1. Semi-Synthetic and Fully Synthetic Heparin-Like Oligosaccharides

The identification of structural determinants in heparin/HS that might mediate the specific binding to a protein would greatly facilitate the design and development of therapeutic agents. Initially this goal was undertaken by mapping “minimal binding sequences” in heparin/HS utilizing fragments of the polysaccharides generated by enzymatic digestion or by testing chemically modified GAGs with different sulfate content [47,48,49]. The heterogeneous nature of these preparations has ultimately limited their usefulness in protein binding studies. Their use has however concurred to assess the criticality of certain sulfate residues in heparin for binding to a given protein. The objective of identifying short high-affinity binding oligosaccharides is currently pursued using panels of structurally defined HS-like oligosaccharides generated through chemoenzymatic or completely synthetic processes [50,51]. An example of this approach concerns the investigation of the role of 6O-sulfation in HS-oligosaccharides. The sulfation at this position accelerates the formation of the ternary complex with FGF and FGFR1 and promotes signal transduction [52,53,54]. In dodecasaccharides composed of alternating 2-O-sulfated iduronate and N-sulfated glucosamine, the addition of a single 6-OS at the non-reducing end of the chain did not affect the ability of the oligosaccharide to compete with heparin and form a tripartite complex with FGF2 and FGFR1 but had negative impact on FGF2 signaling (Figure 2B) [55]. When tested in a cell system, the single-6O-sulfated dodecasaccharide inhibited FGF2-mediated endothelial cell proliferation, migration and sprouting in vitro, exceeding in this the effect of the parent oligosaccharide. On the other hand, VEGF-dependent endothelial cell functions and angiogenesis were not inhibited by any of the oligosaccharide tested. In another study the structural requirements of tetra-oligosaccharides for binding to FGF1 and FGF2 in complex with FGFR1c2 and to members of the CCL and CXCL chemokines families has been investigated [56]. While the position of the sulfated groups along the chain and their frequency clearly influenced the binding affinity, no particular sequence could provide a sufficient degree of binding specificity needed to engage a single protein. These findings underscore the intrinsic difficulty that is encountered in the development of heparin-like oligosaccharides acting on a single protein target or with a sufficient spectrum of activity required to block angiogenesis. 

### 2.2. Non-Carbohydrate Small Molecules as FGF Antagonists

**Figure 3:**
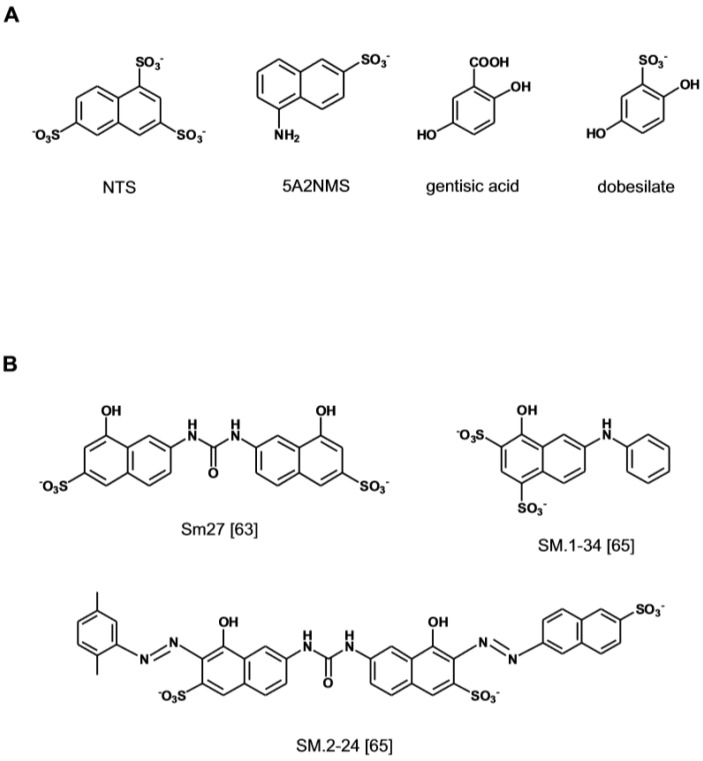
Non-carbohydrate small molecules binding to the heparin-binding site of FGF1 identified by screening of focused chemical libraries and optimized through rational design. (**A**) Ligands based on the 1,3,6-naphtalenetrisulfonate (NTS) lead. (**B**) Small molecule ligands based on the TSP1-FGF2 interaction model. Structures redrawn from refs. [63,65].

Early structure–activity studies had highlighted the importance of the naphtalene sulfonate moiety 1,3,6-naphtalenetrisulfonate (NTS) for the anti-angiogenic activity of suramins and suradistas [39,57] (Figure 3A). It was later established that NTS embodies structural features that enable its interaction with the heparin binding site of FGF1 [58]. A search of anti-angiogenic compounds based on the NTS scaffold has led to the identification of 5-amino-2-naphtalensulfonate (5A2NMS) as a more potent analog. This compound forms a 1:1 molar complex with FGF1 and by docking into the heparin-binding site of the growth factors hinders the access of angiogenic GAGs [59]. A search for other anti-angiogenic molecules utilizing the docking parameters that had been optimized for the naphthalene sulfonates has subsequently lead to the identification of the aspirin metabolite Gentisic acid and its sulfated derivative Dobesilate as FGF1 ligands [60,61]. The compounds inhibit FGF-induced cell proliferation, migration and angiogenesis and display an anti-tumor effect in vivo. The X-ray crystallographic analysis of their complex with FGF1 and the results of competition binding experiments with heparin, indicate that the two small molecules induce structural changes in FGF and FGFR by fitting into the heparin-binding site of the two proteins, thereby inhibiting the formation of the tri-partite complex required for signaling [60]. 

A novel approach to the design of small anti-angiogenic compounds has originated from the structural analysis of the FGF-interacting domain of proteins that modulate FGF activity. Trombosponding-1 (TPS-1) is a FGF2 binding partner that by sequestering the growth factor in the pericellular space inhibits angiogenesis [62]. NMR studies and molecular dynamic simulations have led to the identification of the nature and orientation of the critical chemical elements in the TSP1-1 binding domain for FGF2. A pharmacophore-based approach has subsequently allowed the discovery of non-peptidic small molecules that retain the structural and anti-angiogenic properties of the FGF2 binding sequence of TSP-1 [63] (Figure 3B). The lead compound (SM27) interacts with FGF2 inhibiting the formation of the tripartite complex with angiogenic GAGs and FGFR1 [63,64]. The refinement of the screening algorithm by combining molecular docking simulation and direct binding assay has lately allowed the identification of a new sulfonated bi-naphthalenic agents with higher affinity for FGF2 [65]. 

**Figure 4:**
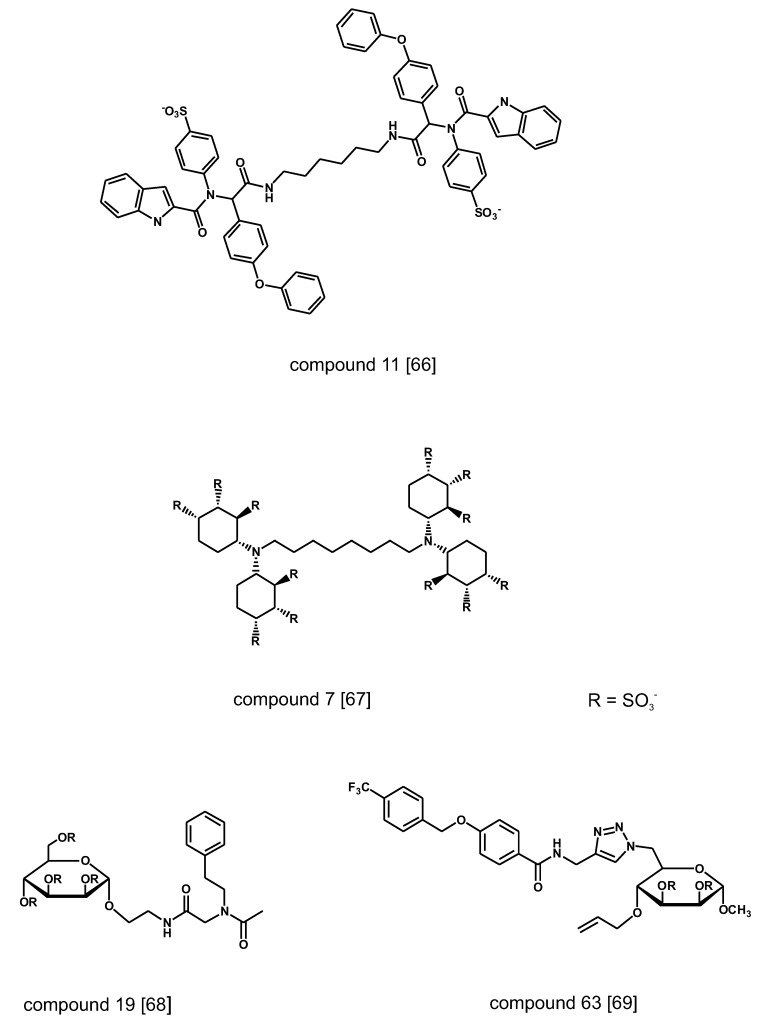
Non-carbohydrate and hybrid small molecules binding to the heparin-binding site of FGF1, FGF2 and VEGF identified by screening of combinatorial chemical libraries. Structures redrawn from refs. [66,67,68,69].

A variety of non-carbohydrate small molecule that compete for binding to VEGF, FGF1 and FGF2 have been discovered by screening chemical libraries of various origin (Figure 4). A combinatorial library of ~19,000 compounds generated by condensation reaction of four-component isocyanides has been screened in a high-throughput (HTS) format using radiolabeled heparin as ligand of FGF2 [66]. The hit compounds have flexible linear structure with bulky hydrophobic moieties at both ends of the chain and negative charges spaced in the neighborhood of 20 A, matching the distance of positive charges in the heparin binding groove of the heparin-binding proteins. In another research program, the binding affinity of a small series of polysulfated cyclitols for FGF1 and FGF2 has been assessed by monitoring by surface plasmon resonance spectroscopy (SPR) the amount of growth factors displaced from immobilized heparin on a chip [67]. The most active compounds display Kd in the nM range. Molecular modeling calculations showed that the sulfate groups of the active compounds overlap with those of heparin in the binding site of FGF2. A follow-up chemical program has centered on a library of sulfated glycoconiugates synthesized by isocyanide-mediated condensation of D-mannopyranoside-derived isocyanides, formaldehyde and a selection of carboxylic acids and amines followed by sulfation of the products [68]. The new compounds displayed lower affinity to FGF2 than cyclitols but could discriminate between FGF1, FGF2 and VEGF with differences up to one order of magnitude in the measured Kd. Similar results have been reported for compounds generated through derivatization via click chemistry of a 6-azido-6-deoxy-a-D-mannopyranoside template [69]. 

## 3. GAG Metabolic Enzymes as Target

**Figure 5:**
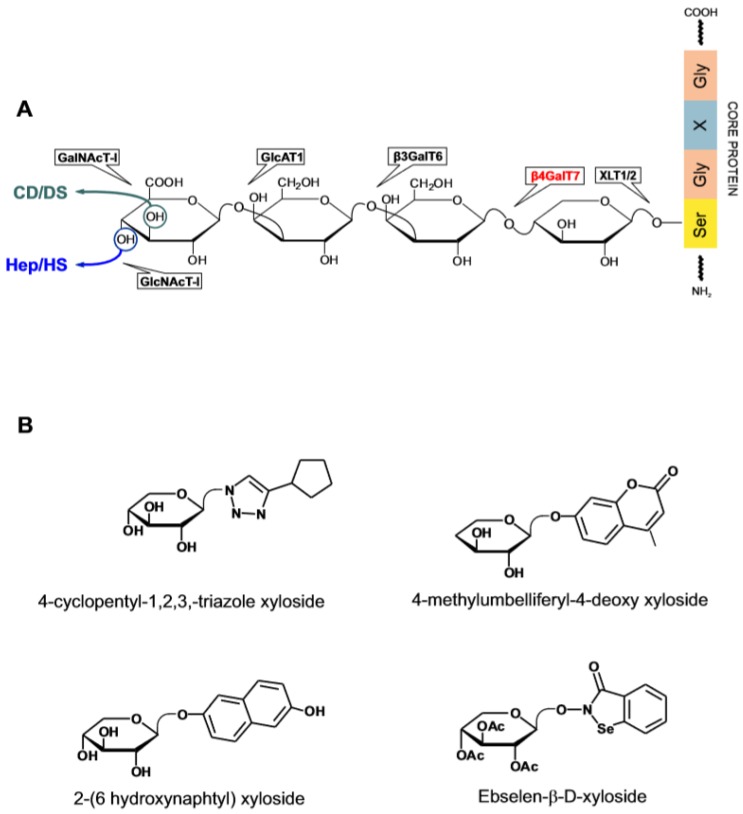
Biosynthesis of the glucosaminoglycans and galactosaminoglycans (GAGs) protein linker tetrasaccharide and recently investigated xylosides. (**A**) Catalytic steps and site of action of β4GalT7. (**B**) Xylosides as Xyl decoy substrates and enzymatic inhibitors of β4GalT7.

Among the best investigated GAG-interacting proteins are the enzymes involved in the metabolism of HS and chondroitin/dermatan sulfate (CS/DS). Sulfated GAGs are secreted as proteoglycans (PGs) anchored to a selected group of proteins [2,24,70]. The glycosylation of a PG core protein begins with the attachment of xylose (Xyl) to serine at a specific position of the polypeptide chain at the time the protein is leaving the endoplasmic reticulum and enters the Golgi apparatus. The addition of this sugar is catalyzed by the GAG chain-initiating enzyme xylosyltransferase (XLT1,2) and is followed by the sequential attachment of two galactose (Gal) residues and D-glucuronic acid (GlcA), each step being mediated by a dedicated enzyme (Figure 5A). The subsequent addition of N-acetylated glucose (GlcNA) or N-acetylated galactose (GalNAc) at the end of the common linker tetrasaccharide commits the biosynthesis to HS or alternatively to CS/DS. A mature GAG chain arises through the action of class-specific enzymes including a set of sulfotransferases acting on the polymerized chain while crossing the Golgi apparatus on its way to secretion [71]. HS-specific enzymes acting extracellularly intervene to change the size and the sulfate content of the secreted polysaccharide. The extracellular HS-editing enzyme heparanase has received particular attention because of its involvement in various pathological states and is a target for drug development. 

### 3.1. Galactosyl Transferase Inhibitors and Decoys

Xylosides (β-D-Xylopyranosides) comprise a family of compounds in which an aglycone is covalently linked to Xyl [72,73]. The effect of xylosides on GAG metabolism has been known for quite some time but the structural determinants affecting their activity have been systematically investigated only recently [74,75,76]. Xylosides can replace Xyl as substrate of the galactosyl transferase β4GalT7—the enzyme catalyzing the formation of the Gal-β-1,4 Xyl bond in the biosynthesis of the tetrasaccharide linker. When a xyloside instead of the protein-bound Xyl is processed by the enzyme, an untethered GAGs chain is generated that is by and large secreted. An elevated cellular permeability afforded by the presence of a non-polar aglycone generally promotes the xyloside priming activity and affects the type of GAG produced. The distance between the sugar and the aglycone, which may be adjusted through the presence of an aliphatic spacers, is likewise an important parameter on which it is possible to intervene to customize the type and the activity of the end-product [77]. Information on the structure of β4GalT7 in complex with the acceptor and donor substrate molecules have led to a further elucidation of the enzymatic mechanism and of the structural requirements for xylosides [77,78,79]. The enzyme has a narrow catalytic pocket which poorly tolerates the presence of substituents on the Xyl ring except when positioned at C4 which is the locus of the catalytic activity. Xylosides modified at this site are a potent inhibitor of the enzyme. The aglycone, on the other hand, sits outside the catalytic pocket and influences the binding rate of the xyloside to the enzyme. 

Because the product of the xyloside activity is a mix of secreted GAGs of different length and variable disaccharide sequence, the profiling of xylosides activity is essentially based on the analysis of the disaccharide composition and the biological activity of the primed GAGs. Notwithstanding these limitations, the reproducible induction of the synthesis of GAGs with a defined biological profile explains the interest in the xylosides as biochemical tools and potential therapeutic agents [73,75,80] (Figure 5B). An example is the ability of benzophenones, benzhydrol and benzhydril β-D-xylosides xylosides administered intravenously or orally to induce the appearance in the bloodstream of anti-thrombotic GAG without triggering the concomitant release of anti-coagulant heparin from mast cells [81,82]. The proposition that suitably designed xylosides can induce cell-specific effects has been recently examined in the context of angiogenesis [83]. Xylosides in which substituted cyclopentane or naphthalene aglycones are conjugated to Xyl through a 1,4-disubsituted-1,2,3-triazole bridge, prime the production by human endothelial cells of GAGs that enhance the pro-angiogenic activity of VEGF and FGF2. These compounds may be useful for treating tissue ischemia and wound healing in which angiogenesis plays a reparative role. Current interest in xylosides includes the design of enzymatic inhibitors of β4GalT7 to block the production of both free and PG-bound GAGs [78]. One of such compounds, 4-Methylumbelliferyl-4-deoxy xyloside lacks the hydroxyl group required for the bond formation with Gal. In primary lung fibroblasts, both constitutive and TGF-β1 stimulated HS and CS/DS PGs production are downregulated by the xyloside [84]. This kind of activity bears promise for the treatment of pulmonary fibrosis which is characterized by excessive matrix PGs deposition under the influence of TGF-β1 activity. 

Whereas xylosides are generally well tolerated by cells even when tested at mM concentration, some display cell-specific cytotoxicity and their mode of action has been elucidated. Thus 2-(6-hydroxynaphtyl)-xyloside is cytotoxic for transformed or tumor-derived cells through a mechanism involving the transport of the primed HS chains to the nucleus [85]. The administration of the xyloside reduced the tumor size from T24 human bladder carcinoma cells implanted subcutaneously in SCID mice by more than 70%. In a follow-up study, 2-naphtyl- or 2-(6-hydroxynaphtyl)-xyloside were shown to prime the production from a breast carcinoma cell line (HCC70) of CS/DS chains that are cytotoxic for the same cells. On the other hand, the GAGs secreted by human breast fibroblasts exposed to the two xylosides had no cytotoxic effect suggesting that the activity of the xylosides is defined by the cell type [86]. β-Xylosides conjugated to linear or cyclic RGD containing peptide that bind to the extracellular α(v)β(3) integrin receptor of activated endothelial and cancer cells have been designed for the purpose of priming GAGs production in specific cell populations [87]. Their excellent priming activity is in line with other findings showing that xylosides can withstand the presence of bulky substituents without losing activity [77]. The recent introduction of drug-derivatized xylosides is a development of this idea. Ebselen is a small molecule seleno-compound that interacts with several enzymes some of which are involved in GAG biosynthesis and is cytotoxic at elevated concentrations [74,88]. In a proof-of-concept study, the cytotoxicity in cancer cells of Ebselen-β-D-xyloside was found to exceed that of Ebselen alone. The apoptotic effect paralleled the increase in the intracellular level of GAGs to which the drug is bound [89]. The authors speculate that by virtue of the anchorage to GAGs, Ebselen is retained inside the cell which causes a prolongation of the cytotoxic effect. Alternatively, Ebselen-conjugated xyloside may prime the production of cytotoxic GAG species.

### 3.2. Sulfotransferase Inhibitors

During the biosynthetic process, the sulfation of the nascent GAG chain is performed by a set of sulfotransferases that decorate the nascent GAG chain with sulfate groups at specific positions. From a teleological point of view, the function of sulfotransferases may be viewed as a process endowing the polysaccharide with specific structural elements required for the high affinity binding to a given protein ligand [90,91,92]. Alternatively, sulfation may be interpreted as the mean to step-wise increase the GAG biological reactivity through the progressive addition of negative charges [5,6]. In the case of coagulation factors and growth factors, the arrangement of the sulfate groups along the polysaccharide chain sensibly affects the binding affinity albeit without necessarily resulting in increased binding specificity [93,94]. In most instances however, the overall negative charge of the polysaccharide rather than the pattern of the sulfate substitutions is the main determinant influencing the binding equilibrium with proteins [21,95]. Research on sulfotransferase inhibitors has been pursued as a means to regulate the structure of neosynthesized GAGs and modulate their interaction with proteins. 

GAG sulfotransferases share a structurally conserved binding site for the sulfate donor PAPS whereas the groove in the catalytic pocket that accommodates the substrate polysaccharide differs [96,97]. A mechanistic equivalence exists between carbohydrate sulfotransferases and protein kinases [98]. The two enzymatic reactions entail the transfer of an anionic group from an adenine-based nucleotide cofactor, either a sulphuryl (PAPS) or a phosphoryl (ATP) donor to their substrates. Crystallographic studies have confirmed that the hydrophobic binding site for PAPS in the catalytic grove of sulfotransferases shares structural similarities with the binding site for adenine in the catalytic pocket of protein phosphokinases [96,99]. This analogy has led to postulate that PAPS binding competitors acting as GAG sulfotransferase inhibitors might bear structural similarities with protein kinases inhibitors acting as ATP binding competitors [98]. This prediction has been confirmed by the recent discovery of sulfotransferase inhibitors by screening chemical libraries of tyrosine kinase inhibitors (TKI) [100]. 

**Figure 6:**
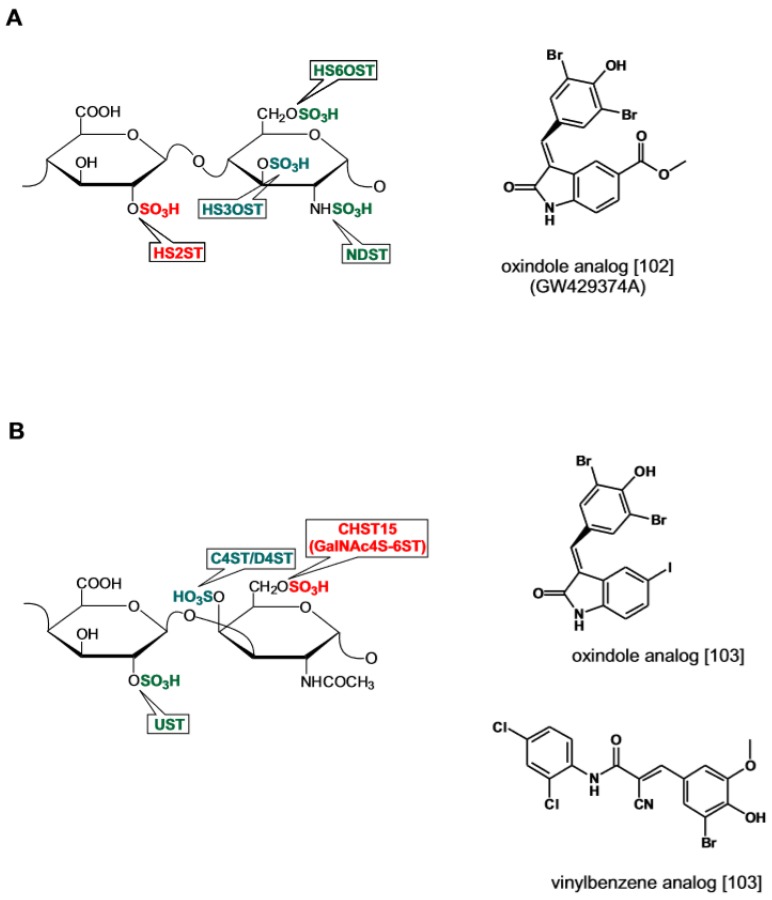
Catalytic activity of sulfotransferases acting on HS and CS and recently identified sulfotransferase inhibitors from screening of tyrosine kinase inhibitors (TKI) and diversity chemical libraries. (**A**) Oxindole HS2ST inhibitor. (**B**) Oxindole and vinylbenzene CHST15 inhibitors. Structures redrawn from refs. [102,103].

During the biosynthesis of sulfated glucosaminoglycans, 2O-sulfotransferase (HS-2OST) catalyzes the sulfation of IdoA and to a lesser extent of GlcA. The enzyme activity locks the uronic acid into its definitive epimeric conformation and as such plays a crucial role in the GAG chain maturation [97,101]. Inhibitors for this enzyme have been identified by screening the Published Kinase Inhibitor Set (PKIS) library comprising 376 kinase inhibitors with a synthetic fluorescent polysaccharide substrate. A group of oxindole c-RAF Ser/Thr kinase inhibitors displayed IC50 values of 20~30 μM toward HS-2OST (Figure 6A) [102]. Another recent study has reported the discovery of inhibitors of sulfotransferase Chst15 responsible for the sulfation of GalNAc at the 6-O position in CS [103]. By screening a library of 70,000 drug-like compounds two were identified that inhibit the enzyme in the low μM range (Figure 6B). The first is a strict analog of the oxindole-based inhibitors acting on HS-2OST. The second displays structural similarity to the EGFR tyrosine kinase inhibitor thyrphostin AG494 featuring a 2-cyano-3-phenyl-*N*-phenylprop-2-enamide scaffold. One of the analogs, compound 34, acts as PAPS competitor and establishes labile covalent bonding with the enzyme cysteines suggesting the possibility that the enzyme redox state is affected. When added to neuronal cells, compound 34 promoted axon extension by inducing the secretion of CSPGs with reduced GlnNAc-6O content in line with the observation that neurite outgrowth is inhibited by GalNAc-6O sulfated CS [104]. Based on these observations the authors foresee the possibility of developing enzymatic inhibitors acting with a high degree of specificity on different classes of sulfotransferases. 

### 3.3. Heparanase Inhibitors

**Figure 7:**
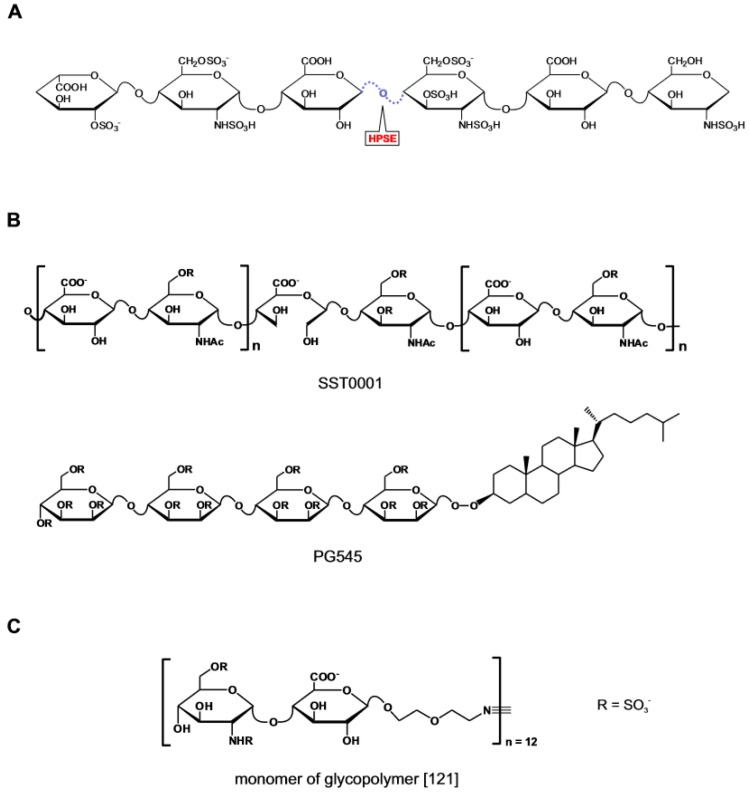
Heparanase inhibitors. (**A**) Schematic representation of the catalytic activity of heparanase acting on heparin/HS. (**B**) Heparanase inhibitors currently in clinical trial. (**C**) Monomeric unit of fully synthetic polymeric inhibitor. Structure redrawn from ref. [121].

Heparanase is a secreted endo-β-D-glucuronidase that cleaves the linkage between GlcNS(6O) and GlcA at selected sites in the HS chain [105] (Figure 7A). The enzyme utilizes an acid catalysis mechanism involving a proton donor and an acceptor identified in Glu225 and Glu334 respectively [106]. Heparanase plays a homeostatic role in the turnover of cellular HS [107,108]. Outside the cell, the enzyme activity reshapes the microenvironment which facilitates tumor cell migration and invasion during cancer metastasis. The pro-tumorigenic activity of the enzyme is mediated at least in part through the release in the tumor milieu of HS fragments with bound pro-angiogenic factors. During inflammation the heparanase-mediated degradation of the HS component in the extracellular matrix allows the infiltration of pro-inflammatory cells at the site of injury [108]. Considerations such as those that heparanase is the product of a single gene and is upregulated in several human malignant tumors [109], in activated pro-inflammatory cells [110], and in a number of other pathological conditions [111,112], make heparanase an interesting target for drug development [113,114]. 

The structural analogues to heparin and HS have represented the starting point for the discovery of competitive inhibitors of heparanase. To this choice has contributed the availability of heparin surrogates that had been previously generated with the aim of both improving the pharmacological profile and exploring new patenting opportunities in the heparin market [115]. Within this group of compounds, inhibitors of heparanase with lower anticoagulant activity than heparin were selected as candidates for development as anti-tumor agents [13]. The encouraging results from the preclinical studies corroborated the view that a causal link existed between heparanase inhibition and anti-tumor activity. This interpretation has subsequently been re-evaluated in light of the extended spectrum of biological activities displayed by the compounds and the emergence of side-effects during clinical trial [116,117]. Despite this there is still interest to pursuing the clinical development of this class of compounds [118] (Figure 7B). SST0001 (the product of glycol-split N-desulfated and N-acetylated glycol heparin) engages heparanase at sites involved in heparin binding, and allosterically modifies the enzyme [119]. Together with PG545 (an anomerically pure, fully sulfated tetrasaccharide bearing a cholestanol appendage) [120], SST001 is reported in advanced phase of clinical development. A recent addition to this class of compounds is a HS mimetic incorporating structural features introduced based on updated information on the substrate specificity of heparanase [121] (Figure 7C). The twelve-units glycopolymer generated by reacting norbornene-terminated polymerizable monomers each bearing the heparanase substrate GlcNS(6S)α(1,4)GlcA disaccharide, displays stronger anti-heparanase activity than heparin but unlike the latter has no anticoagulant activity and low binding affinity for FGF1, FGF2, VEGF and platelet factor 4. When administered parenterally to mice prior to the inoculation of 4T1 mammary carcinoma cells, the compound inhibited the metastasis of the cells in the breast.

**Figure 8:**
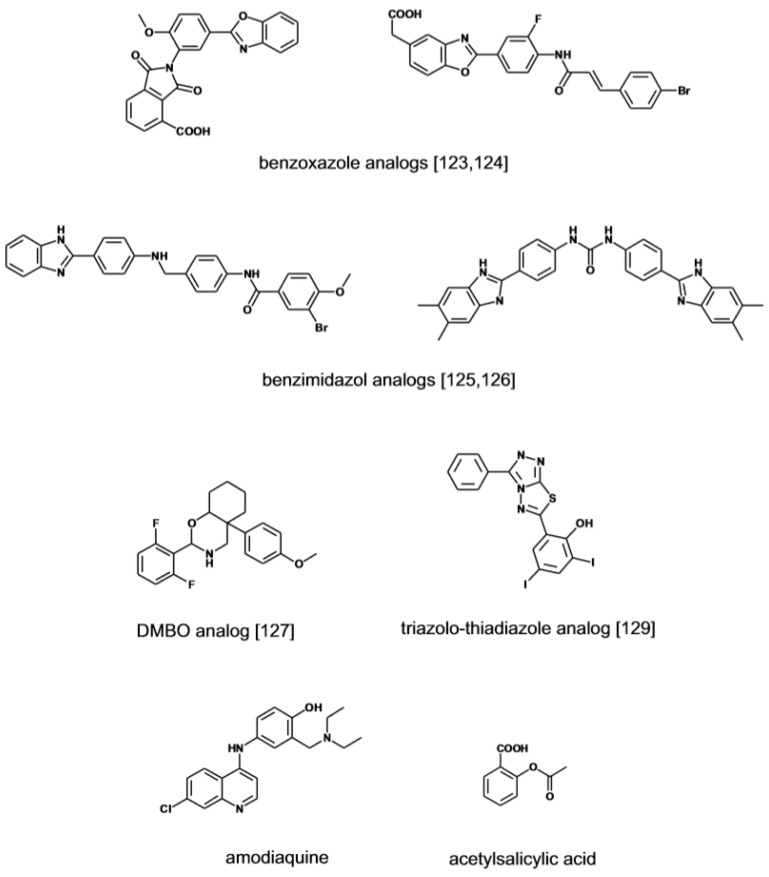
Heparanase inhibitors. Small molecular weight non-carbohydrate heparanase inhibitors. Structures redrawn from refs. [123,124,125,126,127,129].

Small molecular weight heparanase inhibitors structurally unrelated to sulfated polysaccharides have been developed based on the premise that this might afford drugs with focused activity and manageable pharmacodynamic and pharmacokinetic properties. Recently a role has been identified for lysosomal heparanase in enhancing cell autophagy, a process that promotes cell transformation [122]. By virtue of a superior cell penetrating ability, small heparanase inhibitors have the potential compared to high-molecular compounds to effectively neutralize the negative effects of the intracellular enzyme. Compounds screening programs in HTS format have been implemented in search of low molecular inhibitor of the enzyme (Figure 8). A series of 2,3-dihydro-1,2-dioxo-1H-isoindole-5-carboxylic acids bearing a variably substituted benzoxazole moiety were initially identified through this strategy. Further optimization of the leads has yielded inhibitors with activity in the sub μM range [123]. A follow-up program has led to the discovery of furanyl-1,3-thiazol-2-yl and bezoxazolol-5-yl acetic acid derivatives with improved drug-like profile and inhibitory activity [124]. An independent HTS project has identified a series of benzoimidazol-2-yl-benzamides as a novel class of inhibitors with activity in the low to sub μM range [125]. In the effort to further investigate the potential of the scaffold, a series of 4-(1H-benzoimidazol-2-yl)-phenyl-ureas derivatives were prepared yielding compounds with IC50 value in the 0.1 μM range [126]. The screening of small libraries of O-spiroheterocyclic compounds (DMBO), in which an oxazine ring simulates the pyranoside ring structure of HS monosaccharide, has identified heparanase inhibitors with activity in the low μM range [127,128]. The compounds suppress the migration and the invasion of tumor cells but the complex biological activity suggests that in addition to heparanase other proteins are targeted. Lately a group of triazolo-thiadiazole inhibitors has been identified by screening of a small library of heterocyclic compounds [129]. At μM concentrations the inhibitors suppress the migration and invasion of tumor cells across through a Matrigel membrane which is mediated by their anti-heparanase activity. Based on molecular docking calculations, the active compounds bind to heparanase by interacting with key amino acid residues of the catalytic site. Suramin has been among the first reported small molecule inhibitor of heparanase displaying cancer anti-invasive activity in the low μM range [130]. The signature aminonaphtalene sulfonate scaffold of suramin is featured in a new series of analogues [131]. Compared to suramin the compounds display a three-fold increase in heparanase inhibitory activity. A different approach to the development of a specific heparanase inhibitor is pursued with single-stranded nucleic acid aptamers developed through the SELEX protocol [132]. The aptamers bind to heparanase with affinity below the μM mark and dissociate at high ionic strength, suggesting that the heparin-binding site of the enzyme is likely their target. The heparanase-dependent tissue invasiveness of oral cancer cells was inhibited by the aptamers without eliciting cytotoxic effects [133]. 

Other potential inhibitors of the enzyme have emerged from an in-silico screening of a collection of marketed drugs. The strategy in this case has been to generate a pharmacophore model based on the structural analogies shared by known heparanase inhibitors to predict the sites of contact of an hypothetical inhibitor with the enzyme [134]. Following screening of the drug library by molecular docking, the physical interaction of the hit compounds with the enzyme has been directly assessed by biophysical methods (NMR and SPR) and in binding competition experiments using suramin as reference compounds. From this study, the antimalarian drug amodiaquine has emerged as a ligand of the enzyme catalytic site with affinity in the low μM range. 

Non-steroideal anti-inflammatory drugs have long been known as modulators of GAG biosynthesis and activity through unknown mechanisms [74]. In a proteomic study aimed at identify aspirin-bound proteins in cancer cells, heparanase has been detected as a target [135]. MS analysis of the biotinylated drug-enzyme complex recovered by antibody precipitation has revealed that aspirin interacts with amino acids of the catalytic cleft of heparanase that bind the substrate (Lys156) or are directly involved in the β-glucuronidase activity (Glu225) [106]. Aspirin inhibited cell invasion, angiogenesis and tumorigenesis in cell and animal models suitable to test whether the effect observed was secondary to the inhibition of heparanase or alternatively of cyclooxygenase, a primary target for this drug. 

## 4. Heparin-Binding Proteins as Biological Drugs

**Figure 9:**
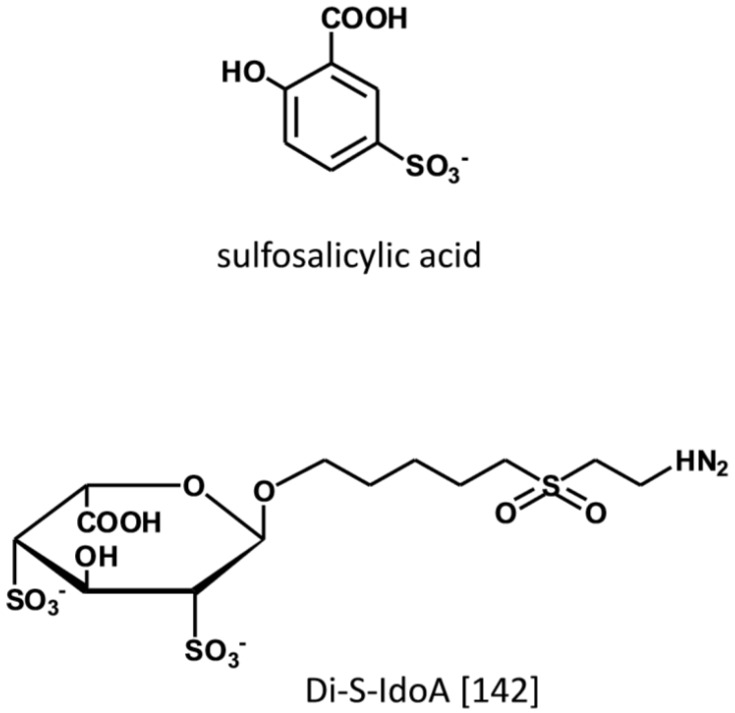
Small molecule interacting with the heparin-binding site of chemokines. Structure redrawn from ref. [142].

During inflammation chemokines in conjunction with GAGs, direct the recruitment, adhesion, and transmigration of leukocytes from the circulation to the site of inflammation [136,137]. By binding to HS present at the luminal side of endothelial cells, chemokines are responsible for the haptotactic and chemotactic gradient that attracts and directs the circulating leukocytes to the site of injury [138,139,140]. Biochemical and biophysical studies demonstrate that GAGs stabilize the formation of chemokines dimers and higher order chemokine oligomers that are required for binding to the transmembrane GPCR chemokine receptor. From the screening by protein NMR of a focused chemical library, non-carbohydrate small molecules have been identified as ligand of the heparin-binding motif in CCL5 with affinity in the μM range (Figure 9) [141]. The lead compound (5-sulfosalicylic acid) inhibits in a dose-dependent manner the cell recruitment in an in-vivo model of induced inflammation with EC50 of 0.06 ug/mouse. Based on measurements of chemical shift perturbations it has been determined that three amino acids within the GAG-binding site of CCL5 in the 40s loop interact with the drug. In another study aimed at identifying ligands to the heparin-binding domain of CCL20, 2,4-O-di-sulfated IdoA (di-S-IdoA) emerged as lead candidate [142]. Mice injected with di-S-IdoA in the tail vein or administered through nasal inhalation had attenuated leukocyte recruitment at the site of inflammation and in the bronchoalveolar lavage fluid consistent with the inhibition of CCL20 activity—a key pro-inflammatory agent in the airways.

Interesting observations have been made by investigating the effect of mutagenizing the GAG-binding site of chemokines. The replacement of positively charged amino acids with neutral amino acids in the HS binding sites of CCL2, CCL5, CCL7, CXCL11 generates heparin-binding deficient proteins that are unable to recruit cells in vivo and furthermore inhibit the activity of the native chemokines in vivo [143,144,145,146]. This effect has been interpreted as the consequence of the inability of heparin-binding deficient chemokines to form active oligomers while retaining the ability to aggregate with native chemokines that are sequestered into non-functional complexes [147]. The anti-inflammatory effect displayed by the heparin-binding deficient chemokines is mirrored by chemokines engineered to enhance their binding to heparin/HS but unable to activate the cognate receptor. The dominant-negative (dn-) agents generated by inserting positively charged amino acids in the heparin-binding domain and by inactivating the receptor-binding function of the native chemokine display anti-inflammatory activity in a variety of conditions [148,149,150]. dn-CCL2 attenuates myocardial ischemia and reperfusion injury in mice by reducing the recruitment of inflammatory monocytes [151]. dn-CCL2 fused to serum albumin to prolong its half-life in serum inhibits the homing in the lung of mice of MC38 tumor cell that synthesizes CCL2 and metastasizes in a CCL2-dependent manner [152]. dn-CXCL8 inhibits trans-endothelial migration of neutrophils by displacing CXCL8 from the surface of endothelial cells. Its anti-inflammatory activity has been investigated in experimental animal models of renal allograft damage, lung inflammation, and arthritis [149]. dn-CXCL12 administered intravenously to mice before the inoculation of human breast cancer cells inhibits the recruitment of the tumor cells by the liver, thus acting as anti-metastatic agent [153]. Similar to what documented for chemokines, the targeted disruption of the heparin-binding site in HGF (hepatocyte-growth factor) and VEGF generates selective competitive antagonists of the parent proteins. Both HGF and VEGF mutants suppress both the normal and oncogenic signaling initiated by the native growth factors in animal models of tumor growth [154]. In other instances, the abrogation of the heparin-binding affinity of a protein has been employed to enhance its tissue permeability and prolong its permanence in circulation. Heparin-binding deficient follistatin fused to the N-terminal of murine IgG1 Fc has improved pharmacokinetics profile and is being developed as systemic agent for muscle regeneration [155]. Collectively the studies endorse the view that the alteration of the heparin-binding site of proteins is a viable strategy for the development of new biodrugs. 

## 5. Summary and Closing Remarks 

The design of heparin/HS inspired drugs with high specificity for proteins remains a daunting task. This is not because structures displaying high affinity for a particular protein cannot be identified. Advances in the synthesis of structurally complex oligosaccharides have been constant and libraries of compounds in quantity suitable for screening are regularly reported. The hard problem is represented by the difficulty to circumvent the cross-reactivity with hundreds of GAG-interacting proteins that are accessible to the oligosaccharide while it is circulating. The high degree of binding complementarity between AT-III and the heparin pentasaccharide is frequently cited as evidence of the possibility of achieving specific glycan–protein interaction through the rational design of GAG-like oligosaccharides. This view however may not capture the exact nature of the specificity at issue and how it comes about. Other heparin pentasaccharide and synthetic sulfated polysaccharides structures can vicariate for the original [156,157,158]. Counterintuitive to the concept of specificity, the pentasaccharide, which is highly represented in heparin, also avidly binds other proteins. In the end, the unique pharmacological properties of the pentasaccharide may be more dependent on the dynamic of the reaction in which it is involved than on the binding specificity. Its peculiarities (high binding affinity to a given protein and rapid and measurable onset of biological activity) may embody the essential requisites necessary for the successful development of other heparin-like oligosaccharides. 

The ability to bind to GAGs is a specialized function that has been acquired independently by different families of proteins during an evolutionary period in which the physicochemical properties and the signature structural motifs of GAGs have substantially remained unchanged [10]. The phylogenetic analysis on two classes of HS-interacting proteins documents the pervasiveness of this adaptive mechanism. In mammals, chemokines CXCL-1, -2 and -3 display close sequence and structure similarities but have distinct biological functions. In a cohort of sixteen mammalian species, this group of chemokines has evolved through gene duplication in a species-specific manner undergoing alterations in the C-terminal coding sequence affecting the charge density of the GAG binding domain. It has been suggested that the different ability to bind to GAGs is at the origin of the chemokines heterogeneity with respect to the formation of concentration gradients under the influence of resident GAGs [159]. Similarly the natural selection process that gave rise to the expanded FGF family tree appears to be based on the diversification of the HS-binding sites that affect the transport of the growth factors in the extra-cellular matrix and the establishment of GAG-dependent concentration gradients [160]. These observations support the notion that heparin-binding sites in chemokines and growth factors critically contribute to the specificity of the functions performed by the different members of these protein families. This paradigm views GAGs as the platform enabling differences in the heparin-binding properties of proteins to become functionally relevant. The finding that heparin-binding sites are not fully conserved in homologous proteins of experimental animals and in humans may invalidate some of the preclinical predictions on the activity a GAG will display when administered to humans [161]. 

Alternative to the design of drugs based on the GAGs scaffold there has been increasing interest in exploring the effect of non-sugar small molecules interacting with heparin-binding site of proteins. In a first group of agents we have included drugs binding to and altering the function of angiogenic factors. The lead molecules have emerged from projects as diverse as ligand-based screening of large chemical libraries, biophysical analysis of the small molecule-protein complex, molecular modeling and structure–activity studies. The examples presented document the variety of approaches tested. In most cases the anti-angiogenic activity of the identified molecules has been confirmed in animal models of disease validating the selection process adopted. Small molecule inhibitors of the enzymes involved in GAG biosynthesis have been developed utilizing information on the structure of the targeted enzymes. Such has been the case for xylosides whose design has taken advantage of information on the conformation of the catalytic complex of β4GalT7. Although mechanistically β4GalT7 cannot be classified as a GAG-binding protein, a digression on this enzyme has given us the opportunity to survey the progress made on the rational design of small molecules drugs interacting with GAG-biosynthetic enzymes. Some xylosides of the new generation display an interesting drug profile. Among the new entries, drug-derivatized xylosides have the potential to orient the pharmacological action of the conjugated drug to cellular subcompartments crossed by GAGs during their biosynthesis and may provide new opportunities for drug repurposing. Analogously, the availability of structural information of the target enzyme has accelerated the discovery of novel HS and CS sulfotransferases inhibitors acting as competitors of the sulfur-donor PAPS. The inclusion of structural features simulating those enabling the substrate GAGs to engage the catalytic site of the targeted enzymes may be necessary to differentiate the structures of the sulfotransferase inhibitors from that of tyrosine-kinase inhibitors that have served as a starting point for the search of PAPS antagonists. For the most part, the heparanase inhibitors have originated from screening of different chemical libraries and only recently attempts have been made to identity a single pharmacophore by analyzing structural features that are shared by the most potent inhibitors. The availability of structural information on the enzyme in complex with its substrate is a recent acquisition and should accelerate the discovery program of ligands of this HS-editing enzyme. Collectively, the studies surveyed document the increasing interest on small molecules targeting GAG-binding proteins with the goal of modulating their function. 

The effect of the direct intervention on the heparin-binding site of proteins through mutagenesis has been investigated as a means to generate biologicals with novel properties. The mutagenized proteins frequently acquire antagonistic activity counteracting the effect of the native entities. This is particularly well documented for a number of chemokines whose function relies on the binding to GAG for the migration at the site of injury and the activation of the signaling receptor. The same strategy has found application for the generation of antagonists of VEGF and HGF that display anti-tumoral activity when tested in animal models. In the case of follistatin, a myocyte growth inducing factor, the abrogation of the heparin-binding property generates a protein with improved pharmacodynamic properties that is under consideration for development as a biological drug. The precise mapping of the heparin-binding sites in proteins and the estimation of their affinity constants is a prerequisite for the continuation of these programs. Techniques such as SPR are routinely applied for this purpose and a coherent picture of the spectrum of proteins competing for binding to GAGs in different tissue districts is beginning to emerge [3,162,163]. Other mapping techniques involve the titration of the heparin-binding sites on proteins by selective labeling. Given the three-dimensional arrangement of multiple heparin-binding sites on the protein surface, the technique allows the identification of those that are primarily involved and others that remain available [164]. The identity of the heparin-binding regions in proteins and their binding affinity are critical piece of information as much as the concentration of the reacting species. Proteins with low binding affinity for heparin if present in plasma at high enough concentration can out-compete high-affinity binding proteins present in low amount [3,8]. Intervening on the heparin-binding property of a given protein is a strategy that can tilt this equilibrium and redirect the system toward a desired outcome. The mode of action of the small molecule and mutagenized proteins we have surveyed may be interpreted in this sense. In view of a possible development as drugs, small molecules are well suited to undergo structural optimization to achieve specificity of action and a desired drug-like profile. The experience with proteins with altered heparin-binding domain encourage further investigation which may generate biological drugs with novel mechanism of action. Advancements in the mapping and profiling of the heparin-binding sites in proteins are well suited to support these efforts.
